# Determinants of Mammographic Breast Density by Race Among a Large Screening Population

**DOI:** 10.1093/jncics/pkaa010

**Published:** 2020-02-26

**Authors:** Justin X Moore, Yunan Han, Catherine Appleton, Graham Colditz, Adetunji T Toriola

**Affiliations:** p1 Division of Public Health Sciences, Department of Surgery, and Siteman Cancer Center at Washington University School of Medicine, St. Louis, MO, USA; p2 Division of Epidemiology, Department of Population Health Sciences, Augusta University, Augusta, GA, USA; p3 Institute of Public and Preventive Health, Augusta University, Augusta, GA, USA; p4 Department of Breast Surgery, First Hospital of China Medical University, Shenyang, Liaoning Province, 110001, China; p5 Breast Imaging Division, Mallinckrodt Institute of Radiology, Washington University in Saint Louis School of Medicine, St. Louis, MO, USA

## Abstract

**Background:**

Because of the mixed reports from smaller studies, we examined associations of race with mammographic breast density and evaluated racial differences in the determinants of breast density.

**Methods:**

Participants included 37 839 women (23 166 non-Hispanic white and 14 673 African American) receiving screening mammograms at the Joanne Knight Breast Health Center at Washington University School of Medicine from June 2010 to December 2015. Mammographic breast density was assessed using the Breast Imaging Reporting and Data System (5th edition). To determine the association of race and participant characteristics with mammographic breast density, we used multivariable polytomous logistic regression models (reference group: almost entirely fatty).

**Results:**

African American women had increased odds of extremely dense (adjusted odds ratio = 1.31, 95% confidence interval = 1.13 to 1.52) and reduced odds of heterogeneously dense breasts (adjusted odds ratio = 0.91, 95% confidence interval = 0.84 to 0.99) compared with non-Hispanic white women. Altogether, race, parity and age at first birth, current age, current body mass index (BMI), BMI at age 18 years, menarche, family history of breast cancer, oral contraceptive use, alcohol use, and menopausal status explained 33% of the variation in mammographic breast density. Among African American and non-Hispanic white women, these factors explained nearly 28.6% and 33.6% of the variation in mammographic density, respectively. Current BMI provided the greatest explanation of breast density (26.2% overall, 22.2% in African American, and 26.2% in non-Hispanic white women).

**Conclusions:**

The determinants of mammographic breast density were generally similar between African American women and non-Hispanic white women. After adjustments for confounders, African Americans had higher likelihood of extremely dense breasts but lower likelihood of heterogeneously dense breasts. The greatest explanation of breast density was provided by BMI, regardless of race.

In the United States, among those diagnosed with breast cancer, the overall survival from breast cancer has increased by more than 21% because of improvements in breast cancer screening practices and treatment modalities (1,2). However, African American women are more likely to be diagnosed with metastatic breast cancer and die from breast cancer compared with non-Hispanic white women (1,3). Mammography screening is associated with a 20% reduction in breast cancer mortality because the early detection of breast cancer reduces the risk of death from the disease (4,5). Thus, mammography screening has great utility in reducing disparities in breast cancer mortality.

Higher mammographic breast density is associated with a four- to sixfold increased risk of breast cancer (6–9). It is estimated that 28 million US women aged 40 to 74 years have dense breasts (10). Mammographic breast density and breast cancer share similar biological and genetic pathways (11,12), and therefore it is important to disentangle the correlates most strongly associated with mammographic breast density and whether they are differential by race. To date, only a few studies have investigated the determinants of mammographic breast density in African American women or whether there are racial differences (13–15), with conflicting results (14). For instance, among women undergoing mammography screening, Oppong et al. (2018) reported that African American women were less likely to have extremely dense breasts when compared with non-Hispanic white women (15), which is in contrast to findings by McCarthy et al. (14), who reported no racial differences in dense breasts when using Breast Imaging Reporting and Data Systems (BI-RADS) but increased odds of having extremely dense breasts among African American women using quantitative breast density measures (14). As a result of these conflicting results, a larger study with a substantial number of African American women is crucial.

To clarify the association of race with mammographic breast density and to determine whether the determinants of mammographic breast density differ by race, we analyzed data from a large population of women undergoing screening mammograms.

## Methods

### Study Design and Population

Study participants were women undergoing screening (annual and biennial) mammograms at the Joanne Knight Breast Health Center at Siteman Cancer Center at Washington University School of Medicine, St. Louis, Missouri, from June 2010 to December 2015. We obtained additional information as part of routine data collection for risk stratification estimation, and investigators returned risk information to women and their providers as part of their care. The Breast Health Center provides mammography services for women from varying socioeconomic and racial backgrounds in the St. Louis region, including those with coverage through breast and cervical cancer screening programs (Centers for Disease Control and Prevention and state funded), the Komen Fund and Barnard Fund coverage for the uninsured, and regularly insured women with private insurance or Medicare coverage. In addition, the Breast Health Center has a breast mammography van that travels throughout the metropolitan St. Louis area to provide mammography services for women within the community setting. For the current study, we excluded women with preexisting cancer (n = 3462); missing information regarding self-reported race and ethnicity (n = 2156); and missing information on mammographic breast density (n = 6226); and those who visit the health center for diagnostics (n = 6740), leaving 37 839 participants included in analyses. The Institutional Review Board at Washington University School of Medicine in St. Louis approved this study (IRB No. 201107282). We obtained informed consent from all participants during visits.

### Mammographic Density Measurements

Women received digital or tomosynthesis mammogram screenings. Qualitative assessment of mammographic density using the American College of Radiology Breast Imaging Reporting and Data System (BI-RADS 5th edition) included almost entirely fatty, scattered areas of fibroglandular density, heterogeneously dense, and extremely dense. We used the most recent mammographic density information and data collected during participants’ latest visit.

### Participant Characteristics

As part of routine breast health services, we collected data on breast cancer risk factors including: age; weight; weight at age 18 years; height; family history of breast cancer; alcohol use; contraceptive use; menopausal hormone use; reproductive characteristics such as age at menarche, parity, and age at first birth; menopausal status; and age at menopause (for postmenopausal women). We identified women as postmenopausal based on self-report of cessation of menstrual periods at the time of mammography. Women were considered postmenopausal if they had undergone bilateral oophorectomy and hysterectomy by the time of mammography. Anthropometric measurements (ie, weight and height) were taken at the time of mammography. We identified whether participants had a first-degree relative (eg, mother or sister) with a history of breast cancer. Average alcohol use over the past year was assessed as nondrinkers, less than 1 drink per month, no more than 1 drink per week, 2–6 drinks per week, and 1 drink per day. Current alcohol use was very low in our study population; hence, we recategorized alcohol consumption into three groups: nondrinkers, current drinkers, and missing. We collected data on oral contraceptive and hormone replacement therapy use. We categorized women as either current, past, or never users based on their self-reported use of oral contraceptives.

### Statistical Analysis

We examined age-adjusted differences in participant characteristics between non-Hispanic white and African American women using χ^2^ tests for categorical variables, *t* tests for parametric continuous variables, and Wilcoxon rank-sum tests for nonparametric continuous variables. We calculated age-adjusted proportions for participant characteristics using indirect standardization (ie, by utilizing the age distribution of our study population). To determine the association of race and participant characteristics with mammographic breast density, we used multivariable polytomous logistic regression models (reference group: almost entirely fatty) and reported odds ratios (ORs) and 95% confidence intervals (CIs). The interpretations of odds ratios derived from polytomous logistic regression are analogous to odds ratios derived from traditional logistic regression and can be interpreted as the log odds of having either extremely dense, heterogeneously dense, or scattered fibroglandular tissue relative to the log odds of having almost entirely fatty (the referent mammographic breast density category) per unit increase or level comparison in a specific covariate (16).

Because some covariates (body mass index [BMI] = 3.0%, BMI at age 18 years = 16.0%, parity and age at first birth = 8.4%, menopause status = 0.4%, family history of breast cancer = 0.4%, alcohol use = 3.2%, age at menarche = 2.6%, and age at menopause = 22.7% [among postmenopausal women only]) were missing, we performed regression modeling with multiple imputations using the fully conditional specification method. When estimating imputed values, we included the following variables in the imputation model: race; age; BMI; BMI at age 18 years; parity and age at first birth; family history of breast cancer; alcohol use; oral contraceptive use; hormone replacement therapy; menopausal status; age at menarche; and age at menopause. We used the fully conditional specification method because we assumed that our underlying data were missing at random, and this approach allows for regression estimation among continuous variables and use of the discriminant function among categorical variables (17,18).

We decided a priori to adjust models for variables that are associated with breast cancer. Therefore, our models were controlled for race; age; BMI; BMI at age 18 years; age at menarche; parity and age at first birth; family history of breast cancer; alcohol use; ever oral contraceptive use; menopausal status; menopausal hormone use (among postmenopausal women only, n* *=* *26 914); and age at menopause (among postmenopausal women only). We assessed collinearity between current BMI and BMI at age 18 years by calculating the correlation and variance inflation factor. There was no evidence of collinearity between current BMI and BMI at age 18 years, because the variance inflation factor was 1.0 and the correlation was 0.49. We repeated these analyses stratified by race; however, we did not include race as a predictor in the race-stratified models. In addition, we examined BMI categories (categorized as <25.0, 25.0–35.0, and >35.0 kg/m^2^) as an effect modifier and repeated main analysis associating race with mammographic breast density stratified by BMI categories. We examined the multiplicative interaction of race and BMI by introducing an interaction variable within our model and present the corresponding *P* value for this association.

To estimate the total variance in mammographic density explained by each explanatory variable, we calculated generalized *R*^2^ values using the Cox and Snell method (19) and the adjusted Nagelkerke method (20) (see Supplementary Table 1, available online). The statistical significance threshold was set to an alpha level of 0.05 for a two-tailed analysis. All statistical analyses were performed using SAS (version 9.4, SAS Institute Inc, Cary, NC) and Stata (version 13, StataCorp LP, College Station, TX).

### Sensitivity Analyses

In sensitivity analyses, we performed logistic regression analyses classifying mammographic breast density into two categories: dense (heterogeneously dense and extremely dense) vs nondense breasts (almost entirely fatty and scattered areas of fibroglandular tissue). We additionally performed analysis with missing categorized as a level of each covariate and presented these within Supplementary Table 2 (available online).

## Results

### Descriptive Results

**Figure 1.**
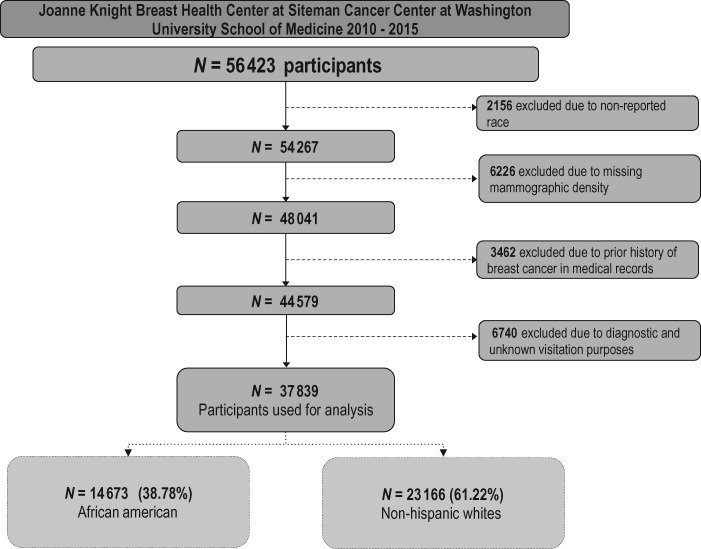
Flowchart of study participants enrolled among women attending the Joanne Knight Breast Health Center at Siteman Cancer Center at Washington University School of Medicine between 2010 and 2015.

A total of 37 839 women were included in this analysis: 14 673 (38.8%) African American and 23 166 (61.2%) non-Hispanic white women. A flowchart depicting inclusion criteria is presented in Figure 1. The African American women were younger (57.1 vs 58.5 years) and had a higher BMI (32.4 vs 28.3 kg/m^2^) compared with the non-Hispanic white women (*P* <.001; Table 1). The African American women had greater parity and were more likely to have their first childbirth before age 25 years (57.5% vs 37.0%), less likely to have a family history of breast cancer in a first-degree relative (15.4% vs 18.2%), less likely to be current alcohol drinkers (35.0% vs 55.6%), and less likely to have ever used menopausal hormone (15.6% vs. 30.2%) (all *P* <.001).

**Table 1.** Age-standardized characteristics among 37 839 women from the Joanne Knight Breast Health Center (BHC) at Siteman Cancer Center, Washington University School of Medicine

Characteristics	Non-Hispanic white (n* *=* *23 166)	African American (n* *=* *14 673)	*P* ^†^
	No. (%) or mean (SD)*	No. (%) or mean (SD)*	
Age, y^‡^ (mean)	58.48 (11.2)	57.14 (10.9)	<.001
Age group, y^‡^ (%)			
<40	306 (1.3)	133 (0.9)	<.001
40–49	4953 (21.4)	3770 (25.7)	
50–59	7913 (34.2)	5289 (36.1)	
60–69	6030 (26.0)	3409 (23.2)	
≥70	3964 (17.1)	2072 (14.1)	
Current BMI in kg/m^2^^‡^ (mean)	28.3 (6.9)	32.4 (7.8)	<.001
BMI at age 18 y in kg/m^2^^‡^ (mean)	21.2 (3.7)	22.1 (4.8)	<.001
Parity, age at first child’s birth^‡^ (%)			
Nulliparous	2960 (12.8)	1283 (8.9)	<.001
1–4 children, <25 y	8571 (37.0)	8472 (57.5)	
1–4 children, 25–29 y	4941 (21.4)	1497 (10.3)	
1–4 children, ≥30 y	3761 (16.2)	897 (6.0)	
≥5 children, <25 y	605 (2.6)	1544 (10.4)	
≥5 children, ≥25 y	146 (0.6)	38 (0.3)	
Missing/not reported	2182 (9.4)	942 (6.7)	
Age at menarche, y^‡^ (mean)	12.7 (1.5)	12.6 (1.8)	<.001
Age at menopause, y^‡^ (mean)	48.7 (5.9)	47.5 (6.6)	<.001
Age at first childbirth, y^‡^ (mean)	25.0 (5.6)	20.8 (5.1)	<.001
Menopausal status (%)			
Premenopause	6482 (29.4)	4357 (27.8)	.001
Postmenopause	16 627 (70.4)	10 287 (72.0)	
Missing/ Not reported	57 (0.2)	29 (0.2)	
Family history of breast cancer			
Yes	4259 (18.2)	2222 (15.4)	<.001
No	18 850 (81.5)	12 422 (84.4)	
Missing/not reported	57 (0.2)	29 (0.2)	
Current alcohol use (%)			
Yes	12 855 (55.6)	5214 (35.0)	<.001
No	9718 (41.8)	9099 (62.4)	
Missing/not reported	593 (2.5)	360 (2.5)	
Oral contraceptive use (%)			
Current	3808 (16.6)	1022 (6.7)	.02
Past	12 067 (52.4)	8463 (57.2)	
Never	7291 (31.0)	5188 (36.1)	
Ever hormone replacement therapy (%)	7166 (30.2)	2176 (15.5)	
BI-RADS density (%)			
1, almost entirely fatty	2389 (10.2)	2537 (17.4)	<.001
2, scattered fibroglandular	12 211 (52.4)	8475 (58.1)	
3, heterogeneously dense	7399 (32.2)	3231 (21.7)	
4, extremely dense	1167 (5.1)	430 (2.8)	

* Presented as N (age adjusted %) or mean (SD). Values are mean (SD) or percentage and are standardized to the age distribution of the study population. Values of polytomous variables may not sum to 100% because of rounding. BI-RADS = Breast Imaging-Reporting and Data System; BMI = body mass index.

^†^ *P* values determined from χ^2^, Wilcoxon rank-sums tests, and *t* tests, as appropriate. All tests were two-sided.

‡ Value is not age adjusted.

### Associations With Breast Density

The odds of having extremely dense breasts (adjusted odds ratio [AOR] = 1.31, 95% CI = 1.13 to 1.52) were higher among African American women, but the odds of heterogeneously dense breasts (AOR = 0.91, 95% CI = 0.84 to 0.99) were lower when compared with non-Hispanic white women (Table 2). Age, current BMI, BMI at age 18 years, parity and age at first birth, and menopausal status were all inversely associated with mammographic breast density. Compared with nulliparous women, parous women, irrespective of age at first birth, had lower odds of extremely dense breasts. The only exception were women with 1–4 children who had their first child after age 30 years. The odds of having extremely dense breasts decreased linearly with age, and we observed the lowest odds ratios among women aged 70 years and older (AOR = 0.14, 95% CI = 0.11 to 0.18) compared with women between 40 and 49 years. We observed reduced odds of extremely dense (AOR = 0.13, 95% CI = 0.12 to 0.14), heterogeneously dense (AOR = 0.36, 95% CI = 0.35 to 0.37), and scattered fibroglandular tissue (AOR = 0.65, 95% CI = 0.63 to 0.67) with every 5-unit increase in BMI kg/m^2^. Similar, but attenuated, associations were observed for BMI at age 18 years. Postmenopausal status was associated with reduced odds of extremely dense breasts (AOR = 0.38, 95% CI = 0.32. to 0.45) compared with premenopausal women.

**Table 2.** Multivariable polytomous logistic regression for the association of participant characteristics with mammographic density (referent outcome is almost entirely fatty) among 37 839 women*

Variable	No. participants^†^	Extremely dense		Heterogeneously dense		Scattered fibroglandular tissue		Almost entirely fatty (referent)
		1597 (4.2)		10 630 (29.1)		20 686 (54.7)		4926 (13.0)
		%^‡^ or mean (SD)	OR (95% CI)	%^‡^ or mean (SD)	OR (95% CI)	%^‡^ or mean (SD)	OR (95% CI)	%^‡^ or mean (SD)
Race								
Non-Hispanic white	23 166	5.0	1.00 (Referent)	31.9	1.00 (Referent)	52.7	1.00 (Referent)	10.3
African American	14 673	2.9	1.31 (1.13 to 1.52)	22.0	0.91 (0.84 to 0.99)	57.8	0.93 (0.87 to 0.99)	17.3
Parity, age at first child’s birth								
Nulliparous	4660	7.0	1.00 (Referent)	33.1	1.00 (Referent)	47.4	1.00 (Referent)	12.5
1–4 children, <25 y	18 526	2.8	0.45 (0.36 to 0.56)	24.4	0.69 (0.60 to 0.80)	58.0	0.99 (0.88 to 1.13)	14.8
1–4 children, 25–29 y	7009	4.8	0.60 (0.47 to 0.76)	32.3	0.85 (0.72 to 1.01)	52.3	1.03 (0.89 to 1.20)	10.7
1–4 children, ≥30 y	5084	7.3	0.87 (0.68 to 1.12)	36.9	1.07 (0.88 to 1.30)	47.7	1.20 (1.01 to 1.44)	8.1
≥5 children, <25 y	2358	1.7	0.27 (0.18 to 0.41)	15.7	0.44 (0.35 to 0.56)	64.3	0.95 (0.81 to 1.13)	18.4
≥5 children, ≥25 y	202	0.8	0.10 (0.01 to 0.77)	28.6	0.76 (0.42 to 1.35)	60.8	1.19 (0.71 to 1.98)	9.8
Age group, y								
<40	439	13.0	1.17 (0.70 to 1.94)	40.3	0.90 (0.59 to 1.38)	38.3	0.83 (0.56 to 1.22)	8.4
40–49	8723	7.3	1.00 (Referent)	36.7	1.00 (Referent)	46.0	1.00 (Referent)	10.0
50–59	13 202	4.1	0.42 (0.35 to 0.50)	28.4	0.51 (0.45 to 0.58)	53.7	0.76 (0.68 to 0.84)	13.8
60–69	9439	2.5	0.23 (0.18 to 0.29)	23.2	0.36 (0.31 to 0.41)	59.7	0.72 (0.63 to 0.81)	14.6
≥70	6036	2.2	0.14 (0.11 to 0.18)	21.7	0.25 (0.21 to 0.29)	62.6	0.63 (0.55 to 0.72)	13.6
BMI^§^	37 839	22.7 (4.2)	0.13 (0.12 to 0.14)	26.3 (5.4)	0.36 (0.35 to 0.37)	30.7 (7.0)	0.65 (0.63 to 0.67)	36.7 (8.6)
BMI at age 18 y^§^	37 839	19.8 (3.2)	0.68 (0.61 to 0.77)	20.4 (3.1)	0.64 (0.60 to 0.68)	21.8 (4.0)	0.82 (0.79 to 0.86)	24.0 (5.4)
Age at menarche, y^‖^	37 839	13.1 (1.6)	1.06 (1.02 to 1.10)	12.9 (1.6)	1.03 (1.01 to 1.06)	12.7 (1.7)	1.01 (0.99 to 1.03)	12.5 (1.7)
FHOBC	6498	5.2	1.77 (1.50 to 2.08)	29.5	1.40 (1.26 to 1.56)	54.2	1.26 (1.15 to 1.38)	11.0
Current alcohol use	18 489	5.4	1.22 (1.06 to 1.41)	32.1	1.15 (1.06 to 1.41)	52.4	1.09 (1.02 to 1.17)	10.2
Oral contraceptive use								
Current	4830	7.1	1.51 (1.23 to 1.86)	37.5	1.37 (1.18 to 1.58)	47.6	1.21 (1.06 to 1.38)	7.8
Past	20 530	3.9	0.96 (0.83 to 1.11)	27.0	0.96 (0.89 to 1.05)	55.5	1.02 (0.95 to 1.09)	13.6
Never	12 479	3.7	1.00 (Referent)	26.2	1.00 (Referent)	56.0	1.00 (Referent)	14.1
Postmenopausal status	26 980	2.9	0.38 (0.32 to 0.45)	24.7	0.54 (0.49 to 0.61)	58.1	0.77 (0.70 to 0.85)	14.3
Ever HRT^¶^	8593	3.6	1.34 (1.07 to 1.67)	27.8	1.15 (1.03 to 1.29)	57.4	1.10 (0.99 to 1.21)	11.3
Age at menopause, y^‖^,^¶^	26 980	48.3 (5.8)	1.02 (1.01 to 1.04)	48.3 (6.1)	1.01 (0.99 to 1.02)	48.2 (6.2)	1.01 (1.01 to 1.01)	47.9 (6.4)

* Fully adjusted for all covariates, model includes race, parity and age at first birth, age group, BMI, BMI at age 18 years, age at menarche, family history of breast cancer, alcohol use, history of oral contraceptive use, and menopausal status. Models among postmenopausal women only further adjusted ever HRT and age at menopause (among postmenopausal women only, n = 26 980). The results from the polytomous logistic regression can be interpreted as the log odds of either extremely dense, heterogeneously dense, or scattered fibroglandular tissue compared with the log odds of almost entirely fatty (the referent mammographic breast density category). Results using fully conditional specification (FCS) multiple imputation. BMI = body mass index; CI = confidence interval; FHOBC = family history of breast cancer; HRT = hormone replacement therapy; OR = odds ratio.

^†^ Approximated strata population derived from multiple imputations.

^‡^ Presented as proportion within variable strata with breast density category. No. (%) represents the total number of participants within strata and proportion of total population.

^§^ BMI and BMI at age 18 years are interpreted as per 5-unit increase.

^‖^ Continuous variables odds ratios are interpreted as per 1-unit increase.

^¶^ Among postmenopausal women only (n = 26 980).

Age at menarche, family history of breast cancer, current alcohol use, and menopausal hormone use were positively associated with mammographic breast density (Table 2). A 1-year increase in age at menarche was associated with 6% increased odds of having extremely dense breasts (AOR = 1.06, 95% CI = 1.02 to 1.10). Women with a family history of breast cancer (AOR = 1.77, 95% CI = 1.50 to 2.08) were at increased odds of extremely dense breasts when compared with women with no family history of breast cancer. Women with current alcohol use (AOR = 1.22, 95% CI = 1.06 to 1.41) were at increased odds of extremely dense breasts when compared with women with no current alcohol use. Among postmenopausal women, ever use of menopausal hormone (AOR = 1.34, 95% CI = 1.07 to 1.67) and older age at menopause (AOR = 1.02, 95% CI = 1.01 to 1.04) were associated with having extremely dense breasts.

Within each race, we examined associated factors for having dense breast using stratified analysis (Table 3). Factors associated with mammographic breast density and the directions of their associations were similar between non-Hispanic white and African American women. However, we observed differential associations by race for family history of breast cancer (AOR = 1.93, 95% CI = 1.59 to 2.36), current alcohol use (AOR = 1.35, 95% CI = 1.12 to 1.61), and hormone replacement therapy (AOR = 1.39, 95% CI = 1.07 to 1.81) among non-Hispanic white women, whereas no association was observed for these factors in African American women.

**Table 3.** Multivariable polytomous logistic regression for the association of participant characteristics stratified by race among 37 839 women*

Variable	No. participants^†^	Extremely dense		Heterogeneously dense		Scattered fibroglandular tissue		Almost entirely fatty
		%^‡^ or mean (SD)	OR (95% CI)	%^‡^ or mean (SD)	OR (95% CI)	%^‡^ or mean (SD)	OR (95% CI)	%^‡^ or mean (SD)
Among African American women								
Total, No. (%)	14 673 (100)	430 (2.9)		3231 (22.0)		8475 (57.8)		2537 (17.3)
Parity, age at first birth								
Nulliparous	1406	5.2	1.00 (Referent)	26.3	1.00 (Referent)	51.6	1.00 (Referent)	17.0
1–4 children, <25 y	8939	2.6	0.48 (0.34 to 0.68)	21.3	0.72 (0.58 to 0.89)	58.8	0.99 (0.83 to 1.19)	17.3
1–4 children, 25–29 y	1657	3.8	0.70 (0.44 to 1.14)	26.9	0.89 (0.67 to 1.19)	52.9	0.91 (0.70 to 1.19)	16.4
1–4 children, ≥30 y	1004	3.8	0.69 (0.41 to 1.16)	28.5	1.03 (0.75 to 1.40)	53.3	1.06 (0.81 to 1.39)	14.4
≥5 children, <25 y	1625	1.6	0.28 (0.17 to 0.48)	13.6	0.43 (0.32 to 0.58)	64.7	0.92 (0.75 to 1.15)	20.1
≥5 children, ≥25 y	42	0.0	Undefined	12.7	0.35 (0.09 to 1.34)	69.8	0.99 (0.41 to 2.40)	17.5
Age group, y								
<40	133	6.8	1.53 (0.59 to 3.97)	33.8	1.20 (0.61 to 2.35)	48.1	1.06 (0.58 to 1.95)	11.3
40–49	3770	5.2	1.00 (Referent)	29.7	1.00 (Referent)	51.1	1.00 (Referent)	14.0
50–59	5289	2.5	0.26 (0.19 to 0.36)	21.5	0.44 (0.37 to 0.52)	57.4	0.71 (0.61 to 0.82)	18.7
60–69	3409	1.8	0.18 (0.12 to 0.27)	17.5	0.33 (0.27 to 0.40)	61.5	0.69 (0.58 to 0.81)	19.2
≥70	2072	1.6	0.10 (0.06 to 0.16)	16.2	0.23 (0.18 to 0.29)	65.3	0.64 (0.53 to 0.78)	16.9
BMI^§^	14 673	24.2 (5.1)	0.15 (0.13 to 0.17)	28.6 (5.8)	0.40 (0.38 to 0.42)	32.6 (7.2)	0.66 (0.64 to 0.69)	38.2 (8.8)
BMI at age 18 y^§^	14 673	20.1 (4.9)	0.80 (0.67 to 0.95)	20.8 (3.7)	0.70 (0.62 to 0.78)	22.1 (4.4)	0.84 (0.80 to 0.89)	24.3 (5.7)
Age at menarche^‖^	14 673	13.1 (1.8)	1.07 (1.01 to 1.14)	12.8 (1.8)	1.02 (0.98 to 1.05)	12.6 (1.8)	1.01 (0.98 to 1.04)	12.5 (1.8)
FHOBC	2228	2.9	1.34 (0.97 to 1.84)	23.4	1.40 (1.19 to 1.65)	58.0	1.22 (1.06 to 1.39)	15.6
Current alcohol use	5356	3.5	1.01 (0.80 to 1.27)	23.8	0.99 (0.88 to 1.12)	57.2	1.04 (0.94 to 1.15)	15.5
Oral contraceptive use								
Current	1022	4.3	1.07 (0.69 to 1.65)	30.0	1.25 (0.97 to 1.62)	52.3	1.07 (0.86 to 1.33)	13.4
Past	8463	2.8	0.93 (0.73 to 1.20)	22.1	1.01 (0.89 to 1.14)	57.5	0.98 (0.88 to 1.08)	17.6
Never	5188	2.9	1.00 (Referent)	20.4	1.00 (Referent)	59.2	1.00 (Referent)	17.5
Postmenopausal status	10 311	2.2	0.57 (0.42 to 0.78)	19.2	0.63 (0.54 to 0.74)	60.2	0.83 (0.73 to 0.96)	18.5
Ever HRT^¶^	2033	2.2	1.18 (0.77 to 1.80)	19.7	1.01 (0.83 to 1.23)	62.1	1.08 (0.93 to 1.26)	16.0
Age at menopause, y,^‖^,^¶^	—	46.3 (6.4)	0.99 (0.96 to 1.02)	47.3 (6.6)	1.00 (0.99 to 1.01)	47.6 (6.4)	1.00 (0.99 to 1.01)	47.5 (6.4)
Among non-Hispanic white women								
Total, No. (%)	23 166 (100)	1167 (5.0)		7399 (31.9)		12 211 (52.7)		2389 (10.3)
Parity, age at first birth								
Nulliparous	3254	7.8	1.00 (Referent)	36.1	1.00 (Referent)	45.6	1.00 (Referent)	10.5
1–4 children, <25 y	9586	3.0	0.44 (0.33 to 0.58)	27.3	0.68 (0.58 to 0.81)	57.3	0.98 (0.84 to 1.16)	12.4
1–4 children, 25–29 y	5353	5.1	0.58 (0.45 to 0.76)	34.0	0.85 (0.70 to 1.03)	52.1	1.09 (0.92 to 1.29)	8.9
1–4 children, ≥30 y	4079	8.2	0.91 (0.68 to 1.21)	39.0	1.09 (0.87 to 1.37)	46.3	1.26 (1.02 to 1.57)	6.5
≥5 children, <25 y	734	1.9	0.27 (0.13 to 0.54)	20.2	0.49 (0.36 to 0.68)	63.4	0.98 (0.75 to 1.28)	14.6
≥5 children, ≥25 years	160	1.0	0.13 (0.02 to 1.01)	32.8	0.91 (0.45 to 1.85)	58.4	1.34 (0.71 to 2.55)	7.8
Age group, y								
<40	306	15.7	0.98 (0.52 to 1.83)	43.1	0.75 (0.43 to 1.30)	34.0	0.68 (0.41 to 1.14)	7.2
40–49	4953	8.9	1.00 (Referent)	42.1	1.00 (Referent)	42.1	1.00 (Referent)	7.0
50–59	7913	5.2	0.54 (0.43 to 0.69)	33.1	0.59 (0.49 to 0.71)	51.3	0.82 (0.69 to 0.97)	10.5
60–69	6030	2.8	0.27 (0.20 to 0.37)	26.4	0.39 (0.32 to 0.48)	58.7	0.75 (0.63 to 0.91)	12.0
≥70	3964	2.5	0.17 (0.12 to 0.23)	24.6	0.26 (0.21 to 0.33)	61.2	0.64 (0.53 to 0.78)	11.8
BMI^§^	23 166	22.2 (3.6)	0.11 (0.10 to 0.13)	25.3 (4.8)	0.34 (0.32 to 0.35)	29.3 (6.6)	0.64 (0.62 to 0.66)	35.0 (8.2)
BMI at age 18 y^§^	23 166	19.6 (2.3)	0.60 (0.51 to 0.70)	20.2 (2.8)	0.58 (0.53 to 0.63)	21.5 (3.7)	0.79 (0.74 to 0.84)	23.6 (5.1)
Age at menarche, y^‖^	23 166	13.1 (1.5)	1.06 (1.00 to 1.12)	12.9 (1.5)	1.04 (1.01 to 1.08)	12.7 (1.5)	1.02 (0.99 to 1.05)	12.4 (1.5)
FHOBC	4271	6.4	1.93 (1.59 to 2.36)	32.7	1.42 (1.23 to 1.63)	52.3	1.29 (1.14 to 1.46)	8.7
Current alcohol use	13 134	6.1	1.35 (1.12 to 1.61)	35.4	1.24 (1.11 to 1.39)	50.4	1.13 (1.02 to 1.25)	8.0
Oral contraceptive use								
Current	3808	7.8	1.65 (1.29 to 2.10)	39.5	1.40 (1.17 to 1.67)	46.4	1.28 (1.09 to 1.51)	6.3
Past	12 067	4.7	0.98 (0.81 to 1.18)	30.5	0.95 (0.85 to 1.07)	54.1	1.06 (0.95 to 1.17)	10.8
Never	7291	4.2	1.00 (Referent)	30.3	1.00 (Referent)	53.8	1.00 (Referent)	11.6
Postmenopausal status	16 669	3.3	0.30 (0.24 to 0.38)	28.1	0.47 (0.40 to 0.56)	56.8	0.70 (0.60 to 0.81)	11.7
Ever HRT^¶^	6560	4.0	1.39 (1.07 to 1.81)	30.3	1.21 (1.05 to 1.40)	55.9	1.11 (0.98 to 1.26)	9.8
Age at menopause, y,^‖^,^¶^	16 669	49.1 (5.3)	1.03 (1.01 to 1.06)	48.7 (5.8)	1.01 (0.99 to 1.03)	48.7 (6.0)	1.01 (0.99 to 1.02)	48.3 (6.3)

* Fully adjusted for all covariates, model includes age at first birth, age group, BMI, BMI at age 18 years, age at menarche, family history of breast cancer, alcohol use, history of oral contraceptive use, and menopausal status. The results from the polytomous logistic regression can be interpreted as the log odds of either extremely dense, heterogeneously dense, or scattered fibroglandular tissue compared with the log odds of almost entirely fatty (the referent mammographic breast density category). Results using fully conditional specification (FCS) multiple imputation. BMI = body mass index; CI = confidence interval; FHOBC = family history of breast cancer; HRT = hormone replacement therapy; OR = odds ratio.

^†^ Approximated strata population derived from multiple imputations.

^‡^ Presented as proportion within variable strata with breast density category. No. (%) represents the total number of participants within strata and proportion of total population.

^§^ BMI and BMI at age 18 years are interpreted as per 5-unit increase.

^‖^ Continuous variables odds ratios are interpreted as per 1-unit increase.

^¶^ Among postmenopausal women only (African American women, n = 10 311; non-Hispanic white women, n = 16 669). Models among postmenopausal further adjusted ever HRT and age at menopause.

We assessed whether BMI categories were effect modifiers on the association between race and mammographic breast density and observed that specifically among overweight women (those with BMI between 25.00 and 34.99 kg/m^2^) there was more than 50% increased odds (AOR = 1.54, 95% CI = 1.13 to 2.10) of having extremely dense breasts comparing African-American with non-Hispanic white women (Table 4). Even among postmenopausal women who had overweight BMI status, African American women were still at 83% increased odds of having extremely dense breasts (AOR = 1.83, 95% CI = 1.19 to 2.10). However, among women with underweight and normal weight BMI status, African American women had more than 35% reduced odds of heterogeneously dense breasts when compared with non-Hispanic white women for both all women (AOR = 0.61, 95% CI = 0.45 to 0.84) and postmenopausal women (AOR = 0.62, 95% CI = 0.44 to 0.88).

**Table 4.** Body mass index (BMI) stratified, multivariable polytomous logistic regression for the association of race with mammographic density (referent outcome is almost entirely fatty) among 37 839 women*

Variable	No. participants^†^	Extremely dense, No. (%)		Heterogeneously dense, No. (%)		Scattered fibroglandular tissue, No. (%)		Almost entirely fatty, No. (%) (Referent)
		1597 (4.2)		10 630 (29.1)		20 686 (54.7)		4926 (13.0)
		%^‡^	OR (95% CI)	%^‡^	OR (95% CI)	%^‡^	OR (95% CI)	%^‡^
BMI categories underweight and normal weight, BMI < 25.0 kg/m^2^^§^								
Race								
Non-Hispanic white	8650	11.4	1.00 (Referent)	47.8	1.00 (Referent)	38.7	1.00 (Referent)	2.1
African American	2249	11.8	0.82 (0.59 to 1.16)	40.3	0.61 (0.45 to 0.84)	44.3	0.75 (0.55 to 1.02)	3.6
Among postmenopausal								
Non-Hispanic white	6031	7.8	1.00 (Referent)	43.8	1.00 (Referent)	45.8	1.00 (Referent)	2.7
African American^‖^	1612	8.8	0.95 (0.65 to 1.40)	36.3	0.62 (0.44 to 0.88)	50.4	0.74 (0.53 to 1.04)	4.4
BMI category overweight, BMI 25.0–30.0 kg/m^2^^§^								
Race								
Non-Hispanic white	6867	2.1	1.00 (Referent)	31.5	1.00 (Referent)	59.1	1.00 (Referent)	7.4
African American	4076	2.8	1.54 (1.13 to 2.10)	29.9	1.01 (0.85 to 1.20)	59.3	1.02 (0.87 to 1.20)	8.0
Among postmenopausal								
Non-Hispanic white	5124	1.2	(Referent)	27.1	(Referent)	62.9	(Referent)	8.8
African American^‖^	2985	1.8	1.83 (1.19 to 2.83)	24.8	1.03 (0.85 to 1.24)	63.8	1.06 (0.89 to 1.26)	9.7
BMI category obese, BMI > 30.0 kg/m^2^^§^								
Race								
Non-Hispanic white	7649	0.5	1.00 (Referent)	14.4	1.00 (Referent)	62.9	1.00 (Referent)	22.2
African American	8347	0.6	1.21 (0.76 to 1.93)	13.3	0.89 (0.79 to 0.99)	60.6	0.87 (0.80 to 0.94)	25.5
Among postmenopausal								
Non-Hispanic white	5514	0.4	1.00 (Referent)	12.0	1.00 (Referent)	63.2	1.00 (Referent)	24.4
African–American^‖^	5714	0.5	1.36 (0.73 to 2.54)	11.4	0.98 (0.85 to 1.13)	61.0	0.93 (0.85 to 1.02)	27.0

* Fully adjusted for all covariates, model includes parity and age at first birth, age group, BMI at age 18 years, age at menarche, family history of breast cancer, alcohol use, history of oral contraceptive use, and menopausal status. The results from the polytomous logistic regression can be interpreted as the log odds of either extremely dense, heterogeneously dense, or scattered fibroglandular tissue compared with the log odds of almost entirely fatty (the referent mammographic breast density category). Results using fully conditional specification (FCS) multiple imputation. BMI = body mass index; CI = confidence interval.

^†^ Approximated strata population derived from multiple imputations.

^‡^ Presented as proportion within variable strata with breast density category. No. (%) represents the total number of participants within strata and proportion of total population.

^§^ *P* value for multiplicative interaction between race and BMI categories <.001.

^‖^ Among postmenopausal women only (underweight and normal weight, n = 7643; overweight, n = 8108; obese, n = 11 229). Models among postmenopausal women only further adjusted ever hormone replacement therapy and age at menopause.

### Variance Explained Results

Combined, the factors in the full model accounted for 33.1% of the variation in mammographic breast density (Supplementary Table 1, available online), with current BMI and BMI at age 18 years independently explaining 26.2% and 9.5%, respectively. Among African American women, the variables included in the model accounted for 28.6% of the variation in mammographic density, with current BMI contributing to 22.2% of the variance. Among non-Hispanic white women, variables in the full model explained 33.6% of the variation in mammographic density, with current BMI accounting for 26.2% of the variance.

### Sensitivity Analysis

Compared with using polytomous logistic regression, the magnitude of the association measures observed while using binary logistic regression (Supplementary Tables 3 and 4, available online) were slightly attenuated, but in the same direction. We also performed analysis with current BMI and BMI at age 18 years as a categorical variable (and missing or not reported as a category) and observed similar effect measures (data not shown). Using complete case analysis (missing categorized as a level for each covariate), we observed similar, but less reliable, estimates (Supplementary Table 2, available online).

## Discussion

In this large study among women undergoing mammographic screening, the determinants of mammographic breast density were similar between African American women and non-Hispanic white women. Age, BMI, age at menarche, parity and age at first birth, menopausal status, family history of breast cancer, alcohol consumption, and menopausal hormone use explained 29% of the variation in mammographic density among African American women and 33% of the variation among non-Hispanic white women, with current BMI accounting for the majority of the variance. African American women had increased odds of extremely dense breasts and reduced odds of heterogeneously dense breasts when compared with non-Hispanic white women. Importantly, BMI was a statistically significant effect modifier on the association between race and mammographic breast density. African American women were more likely to have dense breasts among the overweight population, but less likely to have heterogeneously dense breasts among underweight or normal weight population.

Our findings provide important data on the association of race with mammographic breast density. Prior studies examining racial differences in mammographic density have reported mixed results (13–15,21,22). Some studies reported that African American women have higher breast density compared with non-Hispanic white women, even after adjustments for age, BMI, and reproductive factors (13,21,22). For instance, among women undergoing screening mammograms, El-Bastawissi et al. (2001) (13) reported that African American women were at 30% increased odds of having extremely dense breasts compared with non-Hispanic white women. However, McCarthy et al. reported no differences in dense breasts (categorized as both BI-RADS 3 and 4) by race (14). An important distinction between our study and that of McCarthy et al. is the categorization of BI-RADS groups; McCarthy et al. dichotomized BI-RADS categories into dense and nondense groups, whereas we analyzed BI-RADS as a four-level category. In analyses dichotomizing BI-RADS (using logistic regression), we also observed no association between race and mammographic breast density. Therefore, utilizing polytomous logistic regression allowed for a more robust estimation of effect measures, which enabled us to observe the granularity within the association between race and BI-RADS breast densities. For example, the polytomous logistic regression showed that African American women had increased odds of extremely dense breasts, but reduced odds of heterogeneously dense breasts when compared with non-Hispanic white women. These effects were attenuated when combining heterogeneously dense breasts with extremely dense breasts in the binary logistic regression model. Given that extremely dense and heterogeneously dense breasts (BI-RADS 3 and 4) may be categorized together within studies, it is likely that associations in prior studies examining the relationship between mammographic breast density and cancer outcomes were influenced. Thus, when delineating risks of breast disease by mammographic breast density, it is important to differentiate with as much specificity of BI-RADS groupings as possible.

Consistent with prior research, we observed that, in general, higher BMI was associated with lower mammographic breast density (23). Studies among nationally representative populations report that when compared with non-Hispanic white women, African American women are more likely to have overweight and obese BMI (24–26). Similarly, in this study we observed that African American women had higher BMI than non-Hispanic white women (32.3 vs 28.2 kg/m^2^). Women with higher BMI tend to have larger breasts with more nondense tissue (14,27). Thus, we expected that because the African American women had higher BMI, they would in turn have less dense breasts. Nevertheless, our results showed that African American women were more likely to have dense breasts when compared with non-Hispanic women even after adjusting for BMI and all other potential confounders. After further stratifying by BMI, we observed that specifically among the overweight population, African American women were more than 50% more likely to have dense breasts than non-Hispanic white women. This finding is important as both BMI and breast density are risk factors for morbidity and cancer disease. Therefore, clinicians should take this into account when providing breast mammography services to African American women.

The results of the current study should be interpreted in light of a few inherent limitations. Firstly, because participant characteristics were based on self-reports, they may be subject to misclassification. However, participants were unaware of their breast density during their examination, hence, any misclassification is likely to be nondifferential. Secondly, our imputation analyses assumed that data were missing at random, and although most covariates did not have more than 5% missing, greater than 15% of variables that assessed weight at age 18 years and age at first menopause were missing. Because the imputation analyses predicted missing values based on observed values, our effect estimates should be interpreted with this is mind. However, analysis with missing categories showed slightly attenuated but consistent effect estimates to imputation estimates. Lastly, an inherent limitation of using BI-RADS is that the categorizations are based on two-dimensional images and thus are not sensitive to breast thickness, so may underestimate the true amount of dense tissue in large-breasted women.

Our study has several strengths. To date, our study, with more than 14 000 African American women, is the largest study to examine racial differences in the determinants of mammographic breast density. This allowed for stratified analyses and the ability to observe racial differences. We adjusted for many potential confounders. The Breast Health Center at Washington University School of Medicine provides mammography services to women of diverse socioeconomic status and breast cancer risk profiles and includes women who visited the cancer center and those within the community setting. Thus, our results are generalizable to the larger population of both African American and non-Hispanic white women. We used multinomial-polytomous logistic regression, allowing for a robust statistical approach.

In conclusion, African American women had increased odds of extremely dense breasts but reduced odds of heterogeneously dense breasts compared with non-Hispanic white women. The greatest explanation of breast density was provided by BMI in both African American and non-Hispanic white women.

## Funding

Dr Moore was supported by a training grant from the National Cancer Institute of the National Institutes of Health (award No. T32CA190194). Dr Han was supported by foundations from Barnes-Jewish Hospital and Breast Cancer Research Foundation (award ID: BCRF-17–028). Dr Colditz is supported by the Breast Cancer Research Foundation (award ID: BCRF-17–028). Dr Toriola is supported by grants from the Susan G. Komen Foundation (CCR15332379); NIH/NCI (R21CA216515 and R37CA235602) and Siteman Cancer Center Siteman Investment Program (supported by the Foundation for Barnes-Jewish Hospital Cancer Frontier Fund (BJFH CFF 3781 and 4035) and NCI Cancer Center Support (grant no. P30 CA091842).

## Notes

The content is solely the responsibility of the authors and does not necessarily represent the official views of the National Institutes of Health. The funders had no role in the design of the study; the collection, analysis, and interpretation of the data; the writing of the manuscript; and the decision to submit the manuscript for publication.

Disclaimer: The authors have no conflict of interests to report.

Justin Xavier Moore, Graham Colditz, and Adetunji Toriola conceived and designed the study. Graham Colditz, Catherine Appleton, and Adetunji Toriola oversaw data collection. Justin Xavier Moore and Yunan Han conducted the analysis. Justin Xavier Moore, Yunan Han, Graham Colditz, and Adetunji Toriola contributed to the interpretation of the. Justin Xavier Moore drafted the manuscript and all authors contributed to its critical review.

## Supplementary Material

pkaa010_Supplementary_Data
