# Supplementary material for: Determinants of Mammographic Breast Density by Race Among a Large Screening Population
Source: JNCI Cancer Spectr. 2020 Feb 26;4(2):pkaa010. doi: 10.1093/jncics/pkaa010 (PMC7192029; doi:10.1093/jncics/pkaa010)
Supplement: pkaa010_Supplementary_Data [file pkaa010_supplementary_data.pdf]

SUPPLEMENTARY MATERIALS

Supplementary Methods

To estimate the total variance in mammographic density explained by each explanatory variable, we calculated generalized R^2 values using the Cox & Snell (1989) method [20] (**See Supplemental Table 1**). This model follows the equation:

$$R^2_{Cox\,\&\,Snell} = 1 - \exp\left\{\frac{-2[logL(\beta) - logL(0)]}{n}\right\}$$

, where *L(0)* is the likelihood of the intercept-only model, *L(β)* is the likelihood of the specified model (i.e., model with explanatory variable(s)), and n is the sample size. The $R^2_{Cox\,\&\,Snell}$ cannot attain a value of 1, therefore we used the Nagelkerke (1991) adjustment to obtain "Max-rescaled R^2" values using the following formula:

$$R^2_{Nagelkerke} = \left. 1 - \exp\left\{\frac{-2[logL(\beta) - logL(0)]}{n}\right\} \middle/ 1 - \exp\left\{\frac{-2[logL(0)]}{n}\right\} \right.$$

[21]. We present the R^2 values for both the Cox & Snell (1989) and the adjusted Nagelkerke (1991) in Supplemental Table 1 below.

Supplementary Table 1: Results for R^2 (presented as percentages) values for independently fit models and full models using the polytomous logistic regression.

Variable / Model	Overall*		African American†		White‡	
	$R^2_{Cox\ \&\ Snell}$	$R^2_{Nagelkerke}$	$R^2_{Cox\ \&\ Snell}$	$R^2_{Nagelkerke}$	$R^2_{Cox\ \&\ Snell}$	$R^2_{Nagelkerke}$
Race	2.1	2.3	-	-	-	-
Parity/ age at first birth	3.3	3.7	1.5	1.7	3.0	3.4
Age Group	3.0	3.3	2.4	2.8	3.8	4.3
BMI	23.2	26.2	19.5	22.2	23.2	26.2
BMI at age 18	8.5	9.5	6.7	7.7	8.8	9.9
Age at menarche	0.8	0.9	0.5	0.6	1.0	1.2
FHOBC	0.1	0.2	0.1	0.1	0.2	0.2
Current alcohol use	1.5	1.7	0.3	0.3	1.6	1.8
Oral contraceptive use	1.2	1.3	0.4	0.4	1.1	1.3
Post-menopausal status	2.7	3.1	1.7	1.9	3.8	4.2
Ever HRT§	0.6	0.6	0.1	0.1	0.4	0.5
Age at menopause§	0.1	0.1	0.1	0.1	0.1	0.1
Full Model§	24.6	28.1	21.1	24.3	24.8	28.5
Full Model	29.3	33.1	25.1	28.6	29.7	33.6

* Model among 37,839 women from the Joanne Knight Breast Health Center (BHC) at Siteman Cancer Center. Fully adjusted model contains includes: race, parity and age at first birth, age group, BMI, BMI at age 18, age at menarche, family history of breast cancer, alcohol use, history of oral contraceptive use, menopausal status. Models among post-menopausal women only further adjusted ever HRT and age at menopause (among post-menopausal women only, *N* = 26,914). † Model among 14,673 African American Women. Fully adjusted model includes: parity and age at first birth, age group, BMI, BMI at age 18, age at menarche, family history of breast cancer, alcohol use, history of oral contraceptive use, menopausal status. Models among post-menopausal women only further adjusted ever HRT and age at menopause (among post-menopausal women only, *N* = 10,287). ‡Model among 23,166 Non-Hispanic White Women. Fully adjusted model includes: parity and age at first birth, age group, BMI, BMI at age 18, age at menarche, family history of breast cancer, alcohol use, history of oral contraceptive use, menopausal status. Models among post-menopausal women only further adjusted ever HRT and age at menopause (among post-menopausal women only, *N* = 16,627). §Among postmenopausal women only. The Cox & Snell and Nagelkerke R^2 values are derived using the maximum likelihood function from the fitted models of the polytomous logistic regression models. The Nagelkerke R^2 is the “max rescaled” R^2. They are analogous to the R^2 values derived from the ordinary least squares regression when using linear regression.

Supplementary Table 2: Multivariable polytomous logistic regression* for the association of participant characteristics with mammographic density (referent outcome is almost entirely fatty), among 37,839 women. Model performed with covariates with missing categorized.

		Extremely Dense		Heterogeneously Dense		Scattered Fibroglandular Tissue		Almost Entirely Fatty (Referent)
(%)		1597 (4.2)		10,630 (29.1)		20,686 (54.7)		4926 (13.0)
Variable	No.†	(%‡) or Mean (SD)	OR (95% CI)	(%‡) or Mean (SD	OR (95% CI)	(%‡) or Mean (SD	OR (95% CI)	(%‡) or Mean (SD
Race								
White	23166	5.0	1.00 (Referent)	31.9	1.00 (Referent)	52.7	1.00 (Referent)	10.3
African American	14673	2.9	1.26 (1.07 – 1.49)	22.0	0.91 (0.83 – 0.99)	57.8	0.89 (0.82 – 0.96)	17.3
Parity / age at first child's birth								
Nulliparous	4243	7.1	1.00 (Referent)	33.4	1.00 (Referent)	47.1	1.00 (Referent)	12.4
1 – 4 children, <25 years	17,043	2.6	0.38 (0.31 – 0.48)	23.9	0.63 (0.55 – 0.73)	58.8	0.93 (0.81 – 1.06)	14.7
1 – 4 children, 25 – 29 years	6438	4.5	0.52 (0.41 – 0.66)	32.1	0.81 (0.68 – 0.95)	52.9	0.98 (0.84 – 1.14)	10.6
1 – 4 children, ≥30 years	4658	7.3	0.79 (0.61 – 1.02)	36.7	1.03 (0.85 – 1.25)	47.9	1.17 (0.98 – 1.39)	8.1
≥5 children, <25 years	2149	1.4	0.20 (0.12 – 0.34)	14.8	0.40 (0.32 – 0.51)	65.3	0.94 (0.78 – 1.13)	18.6
≥5 children, ≥25 years	184	0.5	0.10 (0.01 – 0.80)	27.2	0.81 (0.38 – 1.71)	62.0	1.29 (0.65 – 2.56)	10.3
Age group								
<40 years	439	13.0	1.22 (0.70 – 2.13)	40.3	0.97 (0.61 – 2.13)	38.3	0.82 (0.53 – 1.27)	8.4
40 – 49 years	8723	7.3	1.00 (Referent)	36.7	1.00 (Referent)	46.0	1.00 (Referent)	10.0
50 – 59 years	13,202	4.1	0.41 (0.33 – 0.50)	28.4	0.49 (0.42 – 0.56)	53.7	0.76 (0.67 – 0.86)	13.8
60 – 69 years	9439	2.5	0.22 (0.17 – 0.29)	23.2	0.35 (0.30 – 0.41)	59.7	0.72 (0.63 – 0.83)	14.6
70+ years	6036	2.2	0.14 (0.10 – 0.18)	21.7	0.24 (0.20 – 0.28)	62.6	0.62 (0.53 – 0.73)	13.6
BMI§	-	22.6 (4.0)	0.12 (0.11 – 0.13)	26.2 (5.3)	0.35 (0.34 – 0.36)	30.7 (7.0)	0.64 (0.63 – 0.66)	36.8 (8.6)
BMI at age 18§	-	19.7 (3.1)	0.62 (0.55 – 0.70)	20.3 (2.9)	0.58 (0.54 – 0.61)	21.8 (4.0)	0.81 (0.77 – 0.84)	24.1 (5.7)
Age at menarche‖	-	13.1 (1.6)	1.06 (1.02 – 1.11)	12.9 (1.6)	1.02 (0.99 – 1.05)	12.7 (1.7)	1.01 (0.99 – 1.04)	12.5 (1.7)
FHOBC¶	6481	5.2	1.84 (1.55 – 2.20)	29.5	1.39 (1.24 – 1.56)	54.2	1.25 (1.13 – 1.39)	11.1
Current alcohol use	18069	5.4	1.21 (1.04 – 1.40)	32.0	1.13 (1.03 – 1.24)	52.5	1.07 (0.99 – 1.16)	10.2
Oral contraceptive use								
Current	4830	7.1	1.52 (1.22 – 1.91)	37.5	1.41 (1.20 – 1.65)	47.6	1.26 (1.09 – 1.46)	7.8
Ever	20530	3.9	0.98 (0.83 – 1.16)	27.0	0.99 (0.90 – 1.10)	55.5	1.04 (0.96 – 1.13)	13.6
Never	12479	3.7	1.00 (Referent)	26.2	1.00 (Referent)	56.0	1.00 (Referent)	14.1
Post-menopausal status	26914	2.9	0.35 (0.29 – 0.42)	24.7	0.50 (0.44 – 0.57)	58.1	0.71 (0.64 – 0.80)	14.3
Ever HRT#	8575	3.6	1.53 (1.18 – 1.99)	27.8	1.21 (1.06 – 1.39)	57.4	1.14 (1.01 – 1.28)	11.3
Age at menopause‖,#	-	48.5 (5.6)	1.02 (1.01 – 1.04)	48.4 (6.0)	1.01 (1.00 – 1.02)	48.3 (6.2)	1.01 (0.99 – 1.02)	48.0 (6.4)

*Fully adjusted for all covariates, model includes: race, parity and age at first birth, age group, BMI, BMI at age 18, age at menarche, family history of breast cancer, alcohol use, history of oral contraceptive use, menopausal status. Models among post-menopausal women only further adjusted ever HRT and age at menopause (among post-menopausal women only, N = 26,914). The results from the polytomous logistic regression can be interpreted as the log odds of either extremely dense, heterogeneously dense, or scattered fibroglandular tissue compared to the log odds of almost entirely fatty (the referent mammographic breast density category).

†Number of participants per strata. Not reported and missing are coded as a categorical level: Parity / age at first child's birth (missing = 3,124), BMI (missing = 765), BMI at age 18 (missing = 6,047) family history of breast cancer (missing = 86), alcohol use (missing = 953), menopausal status (missing = 83), and age at menarche (missing = 853).

‡ Presented as proportion within variable strata with breast density category.

§ BMI and BMI at age 18 are interpreted as per 5-unit increase.

‖ Continuous variables odds ratios are interpreted as per 1-unit increase.

¶FHOBC = Family history of breast cancer.

#Among post-menopausal women only, N = 26,914. HRT = Hormone replacement therapy.

Supplementary Table 3: Logistic regression for the association between participant characteristics and dense breast*, among 37,839 women. Presented as odds ratios (ORs) and associated 95% confidence intervals (CIs) for the odds of dense breast. Results using fully conditional specification (FCS) multiple imputation.

	Non-Dense Breast *N* (%) = 25,612 (67.7)	Dense Breast* *N* (%) = 12,227 (32.3)	Model 1†
	N (%)‡ or Mean (SD)§	N (%)‡ or Mean (SD)§	OR (95% CI)
Variable			
Race			
White (Referent)	14600 (63.0)	8566 (37.0)	1.00 (Referent)
African American	11012 (75.1)	3661 (25.0)	1.00 (0.95 – 1.06)
Parity/ age at first birth			
Nulliparous (Referent)	2789 (59.9)	1871 (40.2)	1.00 (Referent)
1 – 4 children, <25 years	13484 (72.8)	5041 (27.2)	0.67 (0.62 – 0.72)
1 – 4 children, 25 – 29 years	4412 (62.9)	2597 (37.1)	0.80 (0.73 – 0.87)
1 – 4 children, ≥30 years	2835 (55.8)	2248 (44.2)	0.89 (0.80 – 0.98)
≥5 children, <25 years	1949 (82.7)	409 (17.4)	0.44 (0.38 – 0.51)
≥5 children, ≥25 years	143 (70.6)	59 (29.4)	0.57 (0.39 – 0.83)
Age group			
<40 years	205 (46.7)	234 (53.3)	1.12 (0.89 – 1.41)
40 – 49 years (Referent)	4886 (56.0)	3837 (44.0)	1.00 (Referent)
50 – 59 years	8910 (67.5)	4292 (32.5)	0.64 (0.60 – 0.69)
60 – 69 years	7017 (74.3)	2422 (25.7)	0.46 (0.42 – 0.50)
70+ years	4594 (76.1)	1442 (23.9)	0.35 (0.32 – 0.39)
BMI‖,¶	31.82 (7.7)	25.82 (5.6)	0.48 (0.47 – 0.50)
BMI at age 18‖,¶	22.20 (4.4)	20.31 (3.1)	0.76 (0.72 – 0.80)
Age at menarche¶	12.61 (1.7)	12.88 (1.6)	1.02 (1.01 – 1.12)
FHOBC #	4242 (65.3)	2256 (34.7)	1.17 (1.10 – 1.25)
Current alcohol use	11571 (62.6)	6919 (37.4)	1.07 (1.01 – 1.12)
Oral contraceptive use			
Current	2677 (55.4)	2153 (44.6)	1.16 (1.07 – 1.26)
Past	14183 (69.1)	6347 (30.9)	0.95 (0.90 – 1.00)
Never	8752 (70.1)	3727 (29.9)	1.00 (Referent)
Post-menopausal status	19529 (72.4)	7451 (27.6)	0.66 (0.62 – 0.71)
Ever HRT**	5900 (68.7)	2692 (31.3)	1.07 (0.99 – 1.15)
Age at menopause¶,**	48.17 (6.2)	48.28 (6.1)	1.00 (0.99 – 1.01)

* Dense breast defined as BI-RADS 3 and 4. Non-dense breast (referent) defined as BI-RADS 1 and 2. †Fully adjusted for all covariates, model includes: race, parity and age at first birth, age, BMI, BMI at age 18, age at menarche, family history of breast cancer, alcohol use, history of oral contraceptive use, menopausal status. ‡Approximated strata population derived from multiple imputations. §Presented as n and column proportions or mean and standard deviations. ‖ Current BMI and BMI at age 18 odds ratios are interpreted as per 5-unit increase (i.e., for every 5 unit increase in BMI there is an associated odds). ¶Continuous variables odds ratios are interpreted as per 1-unit increase (i.e., for every 1 year increase in age at menarche there was a 5% increased odds of having dense breast). # FHOBC = Family history of breast cancer. **Models for age at menopause and HRT are among postmenopausal women only, *N* = 26,980. HRT = Hormone replacement therapy.

Supplementary Table 4: Logistic regression for the association between participant characteristics and the dense breast*, stratified by race 37,839 women. Presented as odds ratios (ORs) and associated 95% confidence intervals (CIs) for the odds of dense breast. Results using fully conditional specification (FCS) multiple imputation.

	Non-Dense Breast *N* (%) = 11,012	Dense Breast* *N* (%) = 3,661	Model 1†
	N (%)‡ or Mean (SD)§	N (%)‡ or Mean (SD)§	OR (95% CI)
Variable			
		Among African American Women (*N* = 14,673)	
Parity/ age at first birth			
Nulliparous (Referent)	963 (68.5)	442 (31.5)	1.00 (Referent)
1 – 4 children, <25 years	6805 (76.1)	2134 (23.9)	0.70 (0.60 – 0.80)
1 – 4 children, 25 – 29 years	1148 (69.4)	508 (30.7)	0.94 (0.79 – 1.12)
1 – 4 children, ≥30 years	680 (67.7)	324 (32.3)	0.94 (0.77 – 1.15)
≥5 children, <25 years	1377 (84.8)	1237 (15.2)	0.44 (0.36 – 0.55)
≥5 children, ≥25 years	37 (87.3)	5 (12.7)	0.31 (0.09 – 1.05)
Age group			
<40 years	79 (59.4)	54 (40.6)	1.16 (0.78 – 1.74)
40 – 49 years (Referent)	2457 (65.2)	1313 (34.8)	1.00 (Referent)
50 – 59 years	4023 (76.1)	1266 (23.9)	0.56 (0.50 – 0.63)
60 – 69 years	2751 (80.7)	658 (19.3)	0.43 (0.37 – 0.49)
70+ years	1702 (82.1)	370 (17.9)	0.31 (0.26 – 0.37)
BMI‖,¶	33.88 (7.9)	28.08 (5.9)	0.52 (0.50 – 0.54)
BMI at age 18‖,¶	22.64 (4.9)	20.71 (3.8)	0.81 (0.74 – 0.89)
Age at menarche¶	12.59 (1.8)	12.80 (1.8)	1.01 (0.99 – 1.04)
FHOBC#	1640 (73.6)	587 (26.4)	1.18 (1.05 – 1.33)
Current alcohol use	3893 (72.7)	1463 (27.3)	1.18 (1.05 – 1.33)
Oral contraceptive use			0.96 (0.88 – 1.05)
Current	671 (65.7)	351 (34.3)	1.16 (0.98 – 1.37)
Past	6360 (75.2)	2103 (24.9)	1.02 (0.93 – 1.11)
Never	3981 (76.7)	1207 (23.3)	1.00 (Referent)
Post-menopausal status	8105 (78.6)	2205 (21.4)	0.73 (0.65 – 0.82)
Ever HRT**	1588 (78.1)	445 (21.9)	0.96 (0.83 – 1.11)
Age at menopause¶,**	47.56 (6.4)	47.17 (6.6)	0.99 (0.98 – 1.01)
		Among Non-Hispanic White Women (*N* = 23,166)	
	Non-Dense Breast *N* (%) = 14,600	Dense Breast* *N* (%) = 8,566	
Parity/ age at first birth			
Nulliparous (Referent)	1825 (56.1)	1429 (43.9)	1.00 (Referent)
1 – 4 children, <25 years	6679 (69.7)	2907 (30.3)	0.66 (0.57 – 0.68)
1 – 4 children, 25 – 29 years	3263 (61.0)	2090 (39.0)	0.66 (0.60 – 0.73)
1 – 4 children, ≥30 years	2155 (52.8)	1924 (47.2)	0.87 (0.77 – 0.97)
≥5 children, <25 years	572 (77.9)	162 (22.1)	0.48 (0.38 – 0.59)
≥5 children, ≥25 years	106 (66.2)	54 (33.8)	0.61 (0.42 – 0.91)
Age group			
<40 years	126 (41.2)	180 (58.8)	1.12 (0.85 – 1.48)
40 – 49 years (Referent)	2429 (49.0)	2524 (51.0)	1.00 (Referent)
50 – 59 years	4887 (61.8)	3026 (38.2)	0.70 (0.64 – 0.77)

60 – 69 years	4266 (70.8)	1764 (29.3)	0.49 (0.44 – 0.54)
70+ years	2892 (73.0)	1072 (27.0)	0.37 (0.33 – 0.42)
BMI[||,¶]	30.27 (7.2)	24.86 (4.8)	0.46 (0.44 – 0.48)
BMI at age 18[||,¶]	21.87 (4.0)	20.14 (2.7)	0.71 (0.67 – 0.76)
Age at menarche[¶]	12.63 (1.5)	12.91 (1.5)	1.03 (1.01 – 1.05)
FHOBC[#]	2602 (60.9)	1669 (39.1)	1.16 (1.07 – 1.26)
Current alcohol use	7678 (58.5)	27277 (41.5)	1.12 (1.05 – 1.19)
Oral contraceptive use			
Current	2006 (52.7)	1802 (47.3)	1.13 (1.03 – 1.24)
Past	7823 (64.8)	4244 (35.2)	0.91 (0.85 – 0.98)
Never	4771 (65.4)	2520 (34.6)	1.00 (Referent)
Post-menopausal status	11423 (68.5)	5246 (31.5)	0.62 (0.57 – 0.68)
Ever HRT[**]	4312 (65.7)	2247 (34.4)	1.11 (1.01 – 1.21)
Age at menopause[¶,**]	48.60 (6.0)	48.75 (5.7)	1.01 (0.99 – 1.02)

[*] Dense breast defined as BI-RADS 3 and 4. Non-dense breast (referent) defined as BI-RADS 1 and 2.

[†] Fully adjusted for all covariates, model includes: age at first birth, age, BMI, age at menarche, family history of breast cancer, alcohol use, history of oral contraceptive use, menopausal status.

[‡] Approximated strata population derived from multiple imputations.

[§] Presented as n and column proportions or mean and standard deviations.

[||] Current BMI and BMI at age 18 odds ratios are interpreted as per 5-unit increase (i.e., for every 5 unit increase in BMI there is an associated odds).

[¶] All continuous variables odds ratios are interpreted as per 1-unit increase (i.e., for every 1 year increase in age at menarche there was a 3% increased odds of having dense breast).

[#] FHOBC = Family history of breast cancer

[**] Models for age at menopause and HRT are among postmenopausal women only (African American Women *N* = 10,310; Non-Hispanic White Women *N* = 16,669). HRT = Hormone replacement therapy,

Supplementary Table 4: Multivariable polytomous logistic regression[*] for the association of participant characteristics with mammographic density (referent outcome is almost entirely fatty), among 37,839 women. Model performed with covariates with missing categorized.

		Extremely Dense		Heterogeneously Dense		Scattered Fibroglandular Tissue		Almost Entirely Fatty (Referent)
(%)		1597 (4.2)		10,630 (29.1)		20,686 (54.7)		4926 (13.0)
Variable	No.[†]	(%[‡]) or Mean (SD)	OR (95% CI)	(%[‡]) or Mean (SD)	OR (95% CI)	(%[‡]) or Mean (SD)	OR (95% CI)	(%[‡]) or Mean (SD)
Race								
White	23166	5.0	1.00 (Referent)	31.9	1.00 (Referent)	52.7	1.00 (Referent)	10.3
African American	14673	2.9	1.26 (1.07 – 1.49)	22.0	0.91 (0.83 – 0.99)	57.8	0.89 (0.82 – 0.96)	17.3
Parity / age at first child's birth								
Nulliparous	4243	7.1	1.00 (Referent)	33.4	1.00 (Referent)	47.1	1.00 (Referent)	12.4
1 – 4 children, <25 years	17,043	2.6	0.38 (0.31 – 0.48)	23.9	0.63 (0.55 – 0.73)	58.8	0.93 (0.81 – 1.06)	14.7
1 – 4 children, 25 – 29 years	6438	4.5	0.52 (0.41 – 0.66)	32.1	0.81 (0.68 – 0.95)	52.9	0.98 (0.84 – 1.14)	10.6
1 – 4 children, ≥30 years	4658	7.3	0.79 (0.61 – 1.02)	36.7	1.03 (0.85 – 1.25)	47.9	1.17 (0.98 – 1.39)	8.1
≥5 children, <25 years	2149	1.4	0.20 (0.12 – 0.34)	14.8	0.40 (0.32 – 0.51)	65.3	0.94 (0.78 – 1.13)	18.6
≥5 children, ≥25 years	184	0.5	0.10 (0.01 – 0.80)	27.2	0.81 (0.38 – 1.71)	62.0	1.29 (0.65 – 2.56)	10.3
Age group								
<40 years	439	13.0	1.22 (0.70 – 2.13)	40.3	0.97 (0.61 – 2.13)	38.3	0.82 (0.53 – 1.27)	8.4
40 – 49 years	8723	7.3	1.00 (Referent)	36.7	1.00 (Referent)	46.0	1.00 (Referent)	10.0
50 – 59 years	13,202	4.1	0.41 (0.33 – 0.50)	28.4	0.49 (0.42 – 0.56)	53.7	0.76 (0.67 – 0.86)	13.8
60 – 69 years	9439	2.5	0.22 (0.17 – 0.29)	23.2	0.35 (0.30 – 0.41)	59.7	0.72 (0.63 – 0.83)	14.6
70+ years	6036	2.2	0.14 (0.10 – 0.18)	21.7	0.24 (0.20 – 0.28)	62.6	0.62 (0.53 – 0.73)	13.6
BMI[§]	-	22.6 (4.0)	0.12 (0.11 – 0.13)	26.2 (5.3)	0.35 (0.34 – 0.36)	30.7 (7.0)	0.64 (0.63 – 0.66)	36.8 (8.6)
BMI at age 18[§]	-	19.7 (3.1)	0.62 (0.55 – 0.70)	20.3 (2.9)	0.58 (0.54 – 0.61)	21.8 (4.0)	0.81 (0.77 – 0.84)	24.1 (5.7)
Age at menarche[||]	-	13.1 (1.6)	1.06 (1.02 – 1.11)	12.9 (1.6)	1.02 (0.99 – 1.05)	12.7 (1.7)	1.01 (0.99 – 1.04)	12.5 (1.7)
FHOBC[¶]	6481	5.2	1.84 (1.55 – 2.20)	29.5	1.39 (1.24 – 1.56)	54.2	1.25 (1.13 – 1.39)	11.1
Current alcohol use	18069	5.4	1.21 (1.04 – 1.40)	32.0	1.13 (1.03 – 1.24)	52.5	1.07 (0.99 – 1.16)	10.2
Oral contraceptive use								
Current	4830	7.1	1.52 (1.22 – 1.91)	37.5	1.41 (1.20 – 1.65)	47.6	1.26 (1.09 – 1.46)	7.8
Ever	20530	3.9	0.98 (0.83 – 1.16)	27.0	0.99 (0.90 – 1.10)	55.5	1.04 (0.96 – 1.13)	13.6
Never	12479	3.7	1.00 (Referent)	26.2	1.00 (Referent)	56.0	1.00 (Referent)	14.1
Post-menopausal status	26914	2.9	0.35 (0.29 – 0.42)	24.7	0.50 (0.44 – 0.57)	58.1	0.71 (0.64 – 0.80)	14.3
Ever HRT[#]	8575	3.6	1.53 (1.18 – 1.99)	27.8	1.21 (1.06 – 1.39)	57.4	1.14 (1.01 – 1.28)	11.3
Age at menopause[||,#]	-	48.5 (5.6)	1.02 (1.01 – 1.04)	48.4 (6.0)	1.01 (1.00 – 1.02)	48.3 (6.2)	1.01 (0.99 – 1.02)	48.0 (6.4)

[*]The results from the polytomous logistic regression can be interpreted as the log odds of either extremely dense, heterogeneously dense, or scattered fibroglandular tissue compared to the log odds of almost entirely fatty (the referent mammographic breast density category). Models are fully adjusted for all covariates, model includes: race, parity and age at first birth, age group, BMI, BMI at age 18, age at menarche, family history of breast cancer, alcohol use, history of oral contraceptive use, menopausal status. Models among post-menopausal women only further adjusted ever HRT and age at menopause (among post-menopausal women only, N = 26,914).

[†] N (%) – represents the total number of participants within strata and proportion of total population. Not reported and missing are coded as a categorical level: Parity / age at first child's birth (missing = 3,124), BMI (missing = 765), BMI at age 18 (missing = 6,047) family history of breast cancer (missing = 86), alcohol use (missing = 953), menopausal status (missing = 83), and age at menarche (missing = 853).

[‡] Presented as proportion within variable strata with breast density category.

[§] BMI and BMI at age 18 are interpreted as per 5-unit increase.

[||] Continuous variables odds ratios are interpreted as per 1-unit increase.

[¶] FHOBC = Family history of breast cancer.

[#] Among post-menopausal women only, N = 26,914. HRT = Hormone replacement therapy.
